# Mitochondria-associated membranes as hubs for neurodegeneration

**DOI:** 10.1007/s00401-015-1528-7

**Published:** 2016-01-07

**Authors:** Michiel Krols, Gert van Isterdael, Bob Asselbergh, Anna Kremer, Saskia Lippens, Vincent Timmerman, Sophie Janssens

**Affiliations:** Peripheral Neuropathy Group, VIB Department of Molecular Genetics, University of Antwerp, Antwerp, Belgium; VIB Department of Molecular Genetics, University of Antwerp, Antwerp, Belgium; Neurogenetics Laboratory, Institute Born Bunge, Antwerp, Belgium; Unit Immunoregulation and Mucosal Immunology, VIB Inflammation Research Centre, University of Ghent, Zwijnaarde, Belgium; Department of Internal Medicine, Ghent University, Ghent, Belgium; Bio Imaging Core, VIB Inflammation Research Center, Ghent, Belgium; VIB Bio Imaging Core, VIB, Ghent, Belgium

## Abstract

There is a growing appreciation that membrane-bound organelles in eukaryotic cells communicate directly with one another through direct membrane contact sites. Mitochondria-associated membranes are specialized subdomains of the endoplasmic reticulum that function as membrane contact sites between the endoplasmic reticulum and mitochondria. These sites have emerged as major players in lipid metabolism and calcium signaling. More recently also autophagy and mitochondrial dynamics have been found to be regulated at ER-mitochondria contact sites. Neurons critically depend on mitochondria-associated membranes as a means to exchange metabolites and signaling molecules between these organelles. This is underscored by the fact that genes affecting mitochondrial and endoplasmic reticulum homeostasis are clearly overrepresented in several hereditary neurodegenerative disorders. Conversely, the processes affected by the contact sites between the endoplasmic reticulum and mitochondria are widely implicated in neurodegeneration. This review will focus on the most recent data addressing the structural composition and function of the mitochondria-associated membranes. In addition, the 3D morphology of the contact sites as observed using volume electron microscopy is discussed. Finally, it will highlight the role of several key proteins associated with these contact sites that are involved not only in dementias, amyotrophic lateral sclerosis and Parkinson’s disease, but also in axonopathies such as hereditary spastic paraplegia and Charcot–Marie–Tooth disease.

**Electronic supplementary material:**

The online version of this article (doi:10.1007/s00401-015-1528-7) contains supplementary material, which is available to authorized users.

## Mitochondria-associated membranes are specialized subdomains of the ER

Compartmentalization of biochemical reactions to dedicated membrane-bound organelles allows eukaryotic cells to perform the plethora of biological processes necessary to maintain homeostasis. While this has clear advantages, it also requires transfer of metabolites and signaling molecules between organelles to maintain cell performance. Such inter-organelle communication is often achieved via vesicular transport or transcriptional pathways. In recent years, alternative pathways involving direct communication between organelles through membrane contact sites (MCSs) have gained much interest. At MCSs, the membranes of two organelles are closely apposed through the formation of protein tethers, enabling fast, direct and reciprocal signaling between both compartments. With distances between the tip of the axon and the cell body reaching up to one meter in humans, the particular morphology of neurons poses limits on vesicular transport and transcriptional pathways. Processes that require strict regulation in space and time are therefore likely to depend much more on such MCSs. We focus this review on the contact sites that are formed between mitochondria and a specialized subdomain of the endoplasmic reticulum (ER) termed Mitochondria-Associated Membranes (MAMs). Emerging evidence shows that dysfunction of the MAMs plays a prominent role in numerous neurodegenerative diseases and that genes affecting ER and mitochondrial homeostasis are clearly overrepresented in hereditary disorders.

**Fig. 1:**
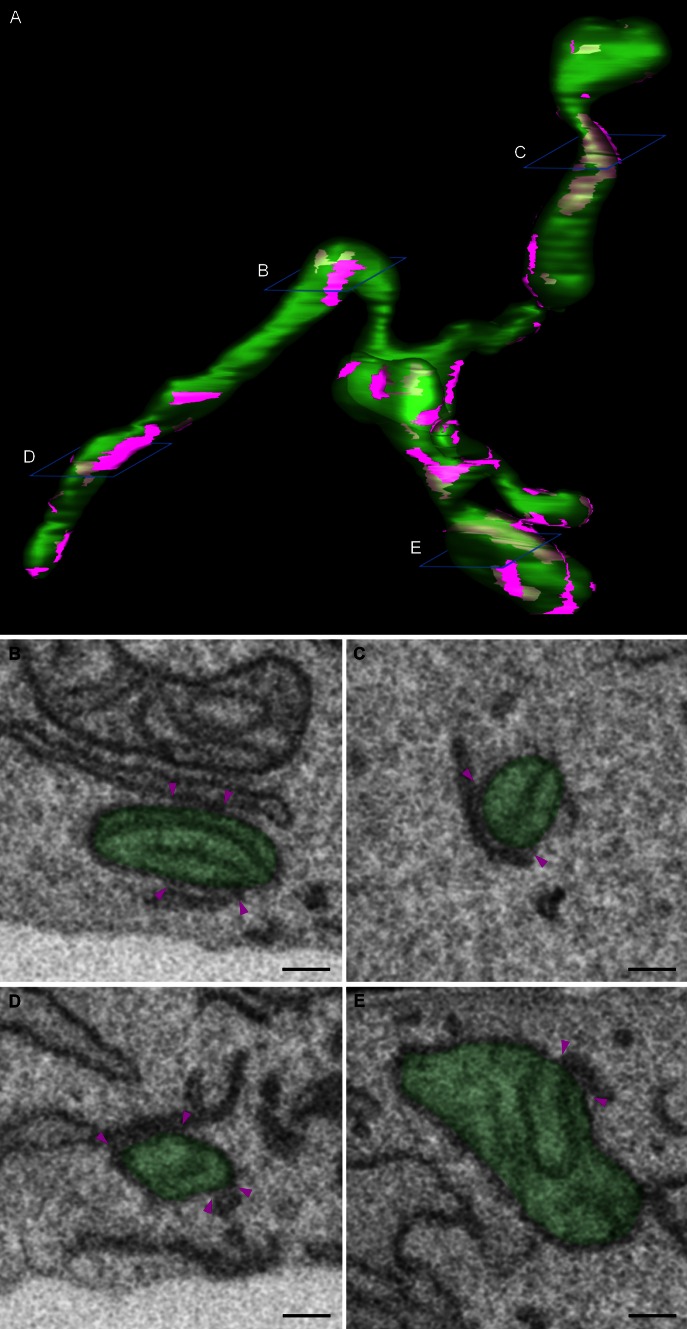
3D morphology of ER-mitochondria contact sites. Mouse embryonic fibroblasts were imaged using a Zeiss Auriga Crossbeam focused ion beam scanning electron microscope (FIB-SEM). By automated serial block face imaging, large image stacks are generated at high resolution, allowing precise 3D reconstruction of a large cellular volume. In this dataset, over 600 10 nm z-slices were obtained resulting in a 3D dataset at 5 × 5 × 10 nm^3^ voxels. Mitochondria and their contact sites with the endoplasmic reticulum (ER) were manually segmented and 3D reconstructed in IMOD (http://bio3d.colorado.edu/imod/). A video of this dataset and the reconstruction can be seen in Supplementary movie 1. **a** The complete reconstruction of two mitochondria (*transparent green*) and their ER-mitochondria contact sites (*magenta*) is shown. A contact site is defined as a region where the ER and the mitochondrial membranes are in closer proximity than 30 nm. It is clear that a single mitochondrion makes multiple contacts with the ER and that these contacts are diverse in size, ranging from punctate sites to large patches of the outer mitochondrial membrane being juxtaposed to the ER. **b**–**e** Represent different examples of scanning electron micrographs extracted from the volume illustrating a section of the mitochondria and their contacts with the ER. The reconstructed mitochondria depicted in **a** are shown in *transparent green*. *Magenta arrowheads* mark the borders of the ER-mitochondria contact sites. The position of these slices is depicted in *blue* in **a**. *Scale bar* 200 nm

Close membrane appositions between the ER and mitochondria have been observed in electron micrographs as early as the 1950s [10, 24]. The ongoing development of subcellular fragmentation techniques repeatedly showed a resilient ER contamination in mitochondrial fractions, substantiating the existence of MCSs between the ER and mitochondria. Only in 1990, with the description of a membrane fraction ‘X’ associated with mitochondria and involved in phospholipid synthesis, the first function for this interface was identified [110]. This fraction ‘X’, now commonly referred to as the MAMs, is a specialized subdomain of the ER with a particular lipid and protein composition that is involved in the crosstalk with mitochondria. These ER-mitochondria contacts have since then been described in several organisms ranging from yeast to mammals [109]. Approximately 100 of such contacts are shown to occur in a single yeast cell and in mammalia approximately 5–20 % of the mitochondrial surface is estimated to be juxtaposed to the ER [1, 88]. Despite the ongoing development of superresolution techniques, the resolution of light microscopy is too limited for precise analysis of the morphology of ER-mitochondria contact sites, with the distance between both membranes at these sites being 10–30 nm. Transmission electron microscopy (TEM) does provide the required resolution; however, even when using tomography, it is very limited in the amount of 3D information which can be acquired. Volume electron microscopy techniques such as focused ion beam scanning electron microscopy (FIB-SEM) recently became available to obtain high-resolution 3D images of a whole cell and can thus be used as a powerful tool to gain more insight into the 3D morphology of MAMs [59]. Figure 1 and Supplementary movie 1 show the 3D reconstruction of mitochondria and their associated MAMs obtained using FIB-SEM imaging of a mouse embryonic fibroblast. These images clearly show that each mitochondrion makes multiple contacts with the ER and that all these contact sites are highly diverse in shape and size. It is currently unknown how MAM morphology relates to its function and therefore volume electron microscopy will be a crucial tool in studying the role different proteins play in regulating these contact sites.

Historically, the ER-mitochondria MCSs have been associated with phospholipid exchange between the ER and mitochondria [88]. In addition, the close proximity between both membranes appears to be critical for the efficient transfer of calcium from the ER to mitochondria. Further studies have revealed additional roles for ER-mitochondria MCSs in a variety of processes ranging from mitochondrial dynamics, sterol metabolism, autophagy, cell survival to energy metabolism and protein folding. Concomitant with the growing appreciation of this signaling hub, the list of disorders associated with ER-mitochondria MCSs is extending. To date this includes cancer [108], metabolic disorders such as Wolfram syndrome [114] or GM1-gangliosidosis [91], diabetes [104], viral infection [48], obesity [7], and neurodegeneration (see Table 1; Boxes 1–4).

**Table 1:**
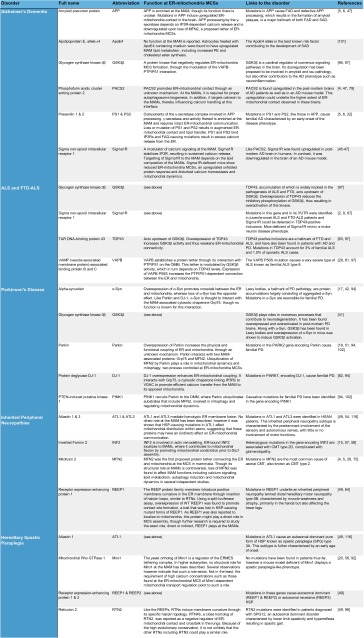
Overview of the genes affected in distinct neurodegenerative diseases and associated with MAM functions and/or structure

Several observations indicate that mitochondria-ER contact sites play crucial roles in neuronal survival and death. Mitochondria in contact with the ER can be readily observed in brain tissue, throughout neurons and at synapses [47]. Already in 2002, it has been suggested that the choreographed interplay between the ER and mitochondria is involved in the shaping of dendritic calcium signals and neuronal activity of hippocampal neurons [84]. In addition at the synapse, calcium shuttling between both compartments was suggested to be essential in determining exocytosis and synaptic activity [74]. The involvement of signaling at the MAMs is implied in several neurodegenerative disorders including Alzheimer’s dementia (AD) [93], Amyotrophic Lateral Sclerosis (ALS) and Frontotemporal Dementia (FTD) [9, 85, 97], Parkinson’s Disease (PD) [17, 42, 82] and Charcot–Marie–Tooth disease (CMT) [28] (for a comprehensive overview see Table 1; Boxes 1–4). More generally, many if not all processes linked to ER-mitochondria contacts are widely implied in neurodegenerative disorders. This review will focus on the structure and function of MAMs and how their disruption contributes to neurodegeneration.

## Structural composition of the MAMs

The MAM fraction can be detached from mitochondria through proteolysis with trypsin or Proteinase K, showing that a proteinaceous tether is responsible for connecting both membranes [26]. The presence of such a protein bridge has been confirmed in electron microscopy studies [26, 97], but despite this, the identity of its components still remains largely elusive. Most proteins that have been found to be enriched at the MAMs, are unlikely to participate directly in tethering, but might be functionally involved in the processes mediated by these contacts [108, 109].

**Fig. 2:**
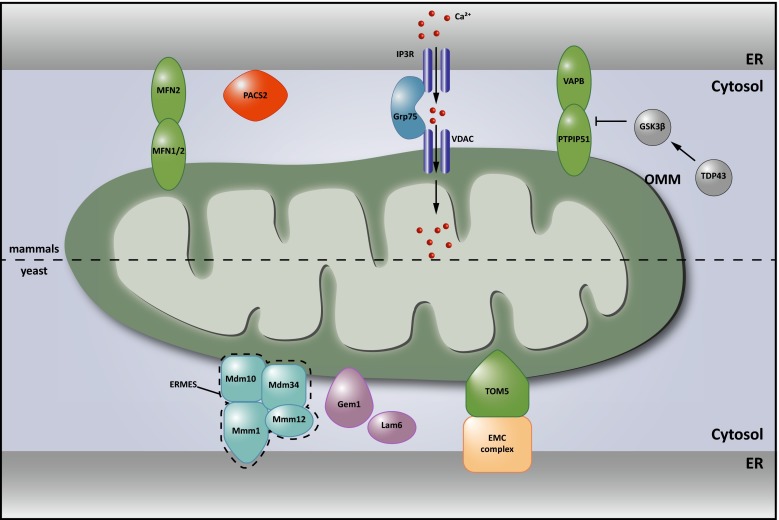
Structural components of ER-mitochondria contact sites. *Upper part* in mammalian cells, dimers between endoplasmic reticulum (ER)-localized Mitofusin (MFN) 2 and mitochondrial MFN1/2 were the first proposed protein tethers. Charcot–Marie–Tooth (CMT)-causing mutations in MFN2 are believed to decrease ER-mitochondrial contact, contributing to the disease. The interaction of VAMP-associated protein B and C (VAPB) in the ER membrane with protein tyrosine phosphatase-interacting protein 51 (PTPIP51) in the outer mitochondrial membrane (OMM) also contributes to anchoring the mitochondria-associated membrane (MAM) to the mitochondrial membrane. This interaction is inhibited by TAR DNA-binding protein 43 (TDP43) in a glycogen synthase kinase 3β (GSK3β) dependent manner. Both these proteins are implied in neurodegeneration (see Table 1; Boxes 1, 2). The amyotrophic lateral sclerosis (ALS)-causing P56S mutation in VAPB on the other hand increases the physical interaction between the ER and mitochondria (Table 1; Box 2). Phosphofurin acidic cluster protein 2 (PACS2) is established as an essential component of these contacts. The levels of PACS2 are found to be altered in the brains of Alzheimer’s dementia (AD) patients (see Table 1; and Box 1). A functional rather than a structural component of the MAMs is formed by a complex between ER-resident inositol 1,4,5-triphosphate (IP3) channels and the mitochondria resident voltage-dependent anion channel (VDAC), which are bridged by Grp75 and important for calcium shuttling between ER and mitochondria (see Fig. 3). *Lower part* in *Saccharomyces cerevisiae*, two tethering complexes are known: the endoplasmic reticulum (ER) mitochondria encounter structure (ERMES), composed of the mitochondrial Mdm10 and Mdm34 proteins, the ER-based Mmm1 protein and the cytosolic Mdm12 protein was the first tether to be described. A second tether important for yeast phospholipid metabolism is achieved through the interaction between the ER membrane complex (EMC) and the outer mitochondrial membrane translocate complex 5 (TOM5). The Miro GTPase Gem1 is a regulatory subunit of ERMES and also Lam6 plays a modulating role determining the extent of membrane contact. It is currently not known whether mammalian orthologs of these components play similar roles in mammalian MAMs

In yeast, genetic screens independently led to the discovery of the ER-Mitochondria Encounter Structure (ERMES) complex [55] and the ER membrane protein complex (EMC) [60] as functional tethers between the ER and mitochondria (see Fig. 2 lower part). The ERMES complex consists of the outer mitochondrial membrane proteins Mdm10 and Mdm34, the ER membrane protein Mmm1 and the cytosolic Mdm12. Loss of any of these proteins could be rescued by the expression of an artificial linker, establishing the structural role of ERMES. Gem1, the yeast ortholog of the mitochondrial Rho GTPases 1 and 2 (Miro1 and Miro2), and more recently also Lam6, were identified as regulators of the ERMES complex [32, 56, 78]. A second screen identified the interaction between the EMC and the outer mitochondrial membrane (OMM) translocase complex 5 (Tom5) as an interaction bridging the ER and mitochondrial membranes independent of ERMES (Fig. 2 lower part). In contrast to the ERMES complex, this contact is essential in the lipid exchange between both organelles [60]. While Lam6, Gem1 and the EMC proteins do have metazoan orthologs, it is as yet unclear whether these orthologs effectively function as regulators of interorganelle contact in mammalia.

The first complex identified in mammalian cells that bridges the gap between the MAM and the OMM is the tripartite complex between the cytosolic chaperone Grp75, the mitochondrial voltage-dependent anion channel 1 (VDAC1) and the inositol 1,4,5-triphosphate receptor (IP3R) in the MAM [100] (Fig. 2 upper part). While knockdown of Grp75 in HeLa cells decreases the transfer of calcium from the ER to mitochondria, ER-mitochondrial linkage is unaffected in cells lacking all three IP3R isoforms [26]. Therefore, rather than representing a structural tether, this interaction is considered a functional one, promoting efficient transfer of calcium from the ER to mitochondria (see below). Other candidate protein tethers are the homodimers or heterodimers of ER-resident Mitofusin (MFN) 2, a dynamin-related GTPase, with MFN1 or MFN2 on the OMM (Fig. 2 upper part) [28]. Cells depleted of MFN2 were reported to have decreased ER-mitochondria contact and crosstalk [5, 28, 44, 82]. CMT-causing mutations in MFN2 failed to rescue the disrupted ER-mitochondria tethering (see Box 4; Table 1) [28]. MFN2 dependent ER-mitochondria crosstalk is tightly controlled by ubiquitination, further supporting its essential role [98]. Still, independent studies could not confirm the crosstalk-promoting role of MFN2 in ER-mitochondria contacts, but rather reported the opposite finding, i.e., loss of MFN2 led to an increase in MCS [25, 34]. These contradictory findings remain to be resolved, as does the effect of CMT-causing mutations in MFN2 on MAM signaling (see Table 1; Box 4).

Adding to this complexity, additional tethering interactions have been identified, which might compensate for MFN2 loss in certain conditions. One such tether is the physical interaction between the MAM protein VAMP (vesicle-associated membrane protein)-associated protein B and C (VAPB) and the mitochondrial protein tyrosine phosphatase-interacting protein 51 (PTPIP51) (Fig. 2 upper part) [29, 97]. Overexpression of either or both of these proteins increases the extent of contact while loss of either protein through siRNA-mediated knockdown significantly diminishes membrane contact and crosstalk [97]. A functional rather than a structural interaction between the ER and mitochondria has been uncovered by establishing the interaction between ER-resident BAP31 and the mitochondrial fission protein Fission 1 homolog (Fis1) [50]. The complex formed between BAP31/Fis1 constitutes an apoptosis-signaling platform needed for the recruitment of procaspase 8, the cleavage of BAP31 and a subsequent increase in Ca^2+^ release from the ER to the mitochondria [50]. This fits with earlier notions that a feedback ER contribution is needed to amplify mitochondrial apoptosis signals [3]. Finally, the vesicular sorting protein phosphofurin acidic cluster sorting protein 2 (PACS2) contributes to the structural integrity of the ER-mitochondria MCSs, through a stabilization mechanism that is not entirely clear [95] (Fig. 2 upper part). The siRNA-mediated knockdown of PACS2 uncouples the ER from mitochondria and induces BAP31 cleavage-dependent mitochondrial fragmentation and cell death in A7 melanoma cells [95], and similarly results in caspase-3 mediated degeneration of primary hippocampal neurons and astrocytes [47]. The complex role of PACS2 as a modulator of MAM properties is discussed further.

Although the mediators of these MCSs seem to be quite distinct between yeast and mammals, the property that different and independent linkers are involved in the coupling of both membranes is conserved. The relevance of having diverse connections is currently unclear, however, it does suggest that these could serve distinct functions. A recent study in mammalian cells indeed indicates the presence of two distinct domains in MAMs that differ in their lipid content and function [4], yet more research is needed to shed light on how distinct protein and lipid compositions can affect MAM function.

## Several essential cellular processes are controlled by MAMs

As stated in the introduction, recent studies revealed several novel functions for the MAMs. In the following sections we will give a brief description of the most important MAM functions in the context of neurobiology.

### Regulation of lipid metabolism

**Fig. 3:**
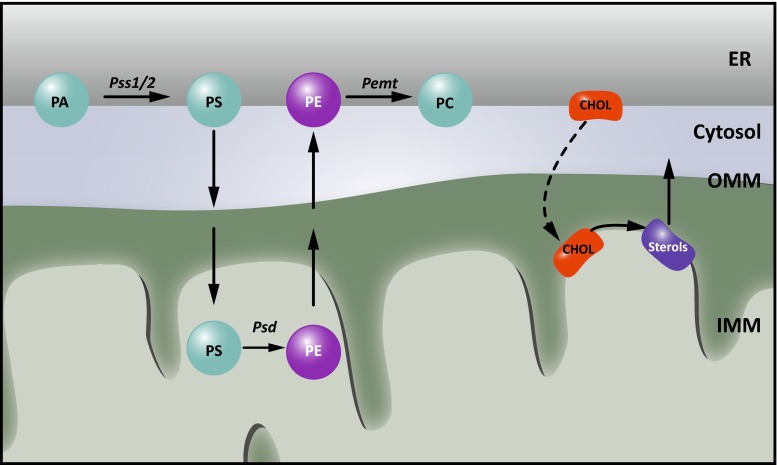
Lipid metabolism at MAMs. Historically, MAMs were identified as essential regions for phospholipid metabolism. For phosphatidylethanolamine (PE) synthesis, phosphatidylserine (PS) synthesized in the endoplasmic reticulum (ER) from phosphatidic acid (PA) by the PS synthetases 1 and 2 (Pss1/2) needs to be shuttled to the inner mitochondrial membrane (IMM), where it is decarboxylated to PE by the PS decarboxylase (Psd). This PE can then be shuttled back to the ER and used in further lipid metabolism, such as conversion to phosphatidylcholine (PC) by the PE-*N*-methyltransferase (PEMT). In addition, the synthesis of sterols requires the import of cholesterol (chol) from the ER into mitochondria

Although most membrane lipid synthesis in eukaryotic cells occurs in the ER, several key metabolic steps and lipid modifications are performed in other organelles, and thus, require transport of lipids. How hydrophobic lipids are distributed within the aqueous environment of the cell towards other organelles remains largely elusive, but recently the role of non-vesicular transport at membrane contact sites has gained a lot of interest [61, 109]. Close membrane apposition at these sites appears to allow efficient shuttling of lipids. In addition, several lipid metabolic enzymes are exclusively localized at certain MCSs, restricting the generation of these lipid species to the site where they are needed. MAMs were originally identified as a fraction highly enriched in phosphatidylserine (PS) synthase-1 and -2 (Pss1 and Pss2) and transfer of phospholipids between the ER and mitochondria was the first function ascribed to ER-mitochondria contacts [109]. Phosphatidylserine, newly synthesized in the MAM by the PS synthases, is subsequently transferred to the closely apposed mitochondrion (Fig. 3). In the inner mitochondrial membrane (IMM), decarboxylation of PS results in the production of phosphatidylethanolamine (PE). The importance of ER-mitochondria MCSs is underlined by the fact that the transfer of PS between both organelles is the rate-limiting step in PE synthesis from PS [112]. In a crude mitochondrial fraction derived from rat liver, newly synthesized PE can then be transferred back to the MAM where it is converted into phosphatidylcholine (PC) by the enzyme PE-*N*-methyltransferase (PEMT) [110]. Since PEMT activity is restricted to hepatocytes, it is not clear how essential MAMs are for PC synthesis in other tissues.

**Fig. 4:**
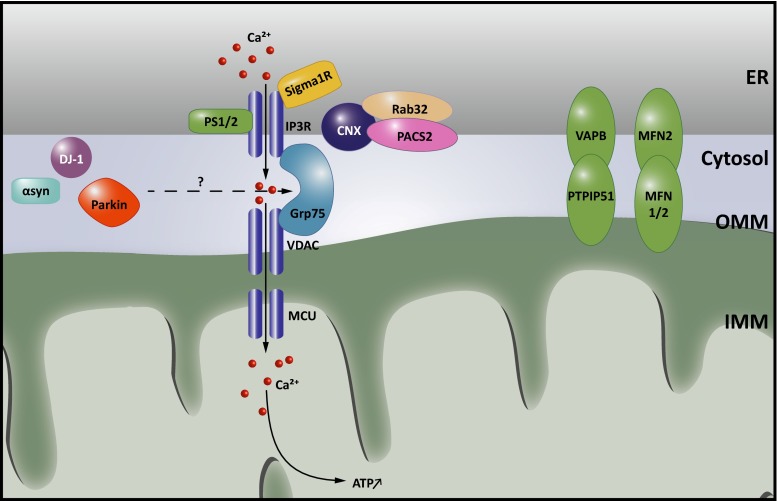
Calcium signaling at ER-mitochondria membrane contact sites. Transfer of calcium to mitochondria requires calcium hotspots, which can be achieved at mitochondria-associated membranes (MAMs) owing to the quasi-synaptic structure of this membrane contact site (MCS). Shuttling of calcium is needed during a variety of responses, as it can both stimulate ATP synthesis and promote mitophagy and apoptosis. Efficient import of calcium at MAMs is mediated by Grp75, which brings the openings of the inositol 1,4,5-triphosphate receptor (IP3R) calcium channels in the endoplasmic reticulum (ER) in close vicinity to the voltage dependent anion channel (VDAC) in the outer mitochondrial membrane (OMM). Opening of the IP3Rs is regulated by a set of proteins present at or recruited to MAMs, including calnexin (CNX), the Sigma non-opioid intracellular receptor 1 (Sigma1R), presenilin 1 and 2 (PS1 and PS2). The presence of CNX at MAMs in turn is regulated by the sorting proteins Rab32 and phosphofurin acidic cluster protein 2 (PACS2), as well as by palmitoylation of CNX. The association of Sigma1R with MAMs on the other hand depends on cholesterol levels. Dysregulation of calcium signaling at MAMs is strongly implied in neurodegenerative disorders. PS1 and PS2 play a complex role regulating both the extent of ER-mitochondrial contact and calcium signaling. The Parkinson’s disease (PD)-associated Parkin, DJ1 and α-synuclein (α-Syn) were also found to affect calcium transfer at these contacts. Finally, defects in mitofusin 2 (MFN2) or VAMP-associated protein B and C (VAPB), which contribute to the structural integrity of the MCSs, affect the calcium crosstalk between the ER and mitochondria

Protein identification approaches revealed that not only Pss1 and Pss2 are enriched at the MAMs, but also several other lipid synthesizing enzymes involved in cholesterol and sphingolipid biosynthesis (Fig. 3) [39, 45, 109]. This fits well with thin layer chromatography studies showing that MAMs comprise a unique lipid profile, with highly elevated levels of ceramides and cholesterol compared to the bulk ER [45]. While it is yet unclear whether the MAMs participate in trafficking of these lipids to mitochondria, this particular lipid raft-like composition plays a determining factor in the recruitment of several well-established MAM components. Both presenilin 1 and 2 (PS1 and PS2), members of the γ-secretase complex are targeted to the MAMs because of their lipid raft composition [106]. Similarly, the chaperone Sigma non-opioid intracellular receptor 1 (Sigma1R), associated with AD, ALS and FTD is strictly dependent on the presence of cholesterol and ceramide for targeting to the MAMs [45] and for performing its essential functions in calcium regulation (see Table 1; Fig. 4).

### Regulation of calcium homeostasis at the MAMs

Already in the 19th century, the role of calcium as second messenger was established through seminal experiments on perfusion of isolated hearts. Since then, a lot of research has been devoted to understand how increases in calcium levels (so-called oscillations) are generated, decoded and translated in signaling events. Two major sources count for the rise in cytosolic calcium: the extracellular medium with a [Ca^2+^] of ~1 mM and the ER with a [Ca^2+^] >100 μM [87]. Mitochondria play a crucial role in buffering increases in cytosolic calcium, and a rise in cytosolic [Ca^2+^]_cyt_ is always closely followed by a rise in [Ca^2+^]_mito_ [89]. Interestingly, upon ER calcium release, mitochondrial levels can rapidly increase 10–100-fold without noticeable rises of cytosolic calcium levels [87]. In contrast to the outer mitochondrial membrane (OMM), which is permeable to calcium ions, influx of calcium into the mitochondrial matrix—driven by the electrical gradient across the IMM—occurs through the recently identified mitochondrial calcium uniporter (MCU) complex. This complex consists of the MCU protein and its regulators (MICU1, MICU2, MCUb, MCUR1, and EMRE) [72]. Paradoxically, the low calcium affinity of the MCU appears incompatible with the observed rapid calcium uptake in mitochondria at low cytosolic calcium levels. This discrepancy was solved by the observation that mitochondria are in close contact with the IP3Rs and ryanodine sensitive channels (RyRs) mediating ER store release [88]. This occurs at the MAMs and leads to the transient formation of “hotspots”, perimitochondrial microdomains in which calcium levels far exceed those in the bulk of the cytosol, enabling rapid import across the IMM [27, 40, 88].

ER-mitochondria MCSs are able to establish such microdomains required for efficient calcium transfer through (1) the close apposition of both membranes, (2) the enrichment of the calcium release channels IP3Rs and RyRs at MAMs and (3) the connection of IP3Rs to the mitochondrial VDAC1 by the chaperone Grp75 (Fig. 4). Knockdown of Grp75 abolishes functional coupling between VDAC1 and IP3R, resulting in diminished calcium transfer [100]. On the other hand, bringing the ER closer to mitochondria through an artificial linker stimulates calcium transfer [26]. Numerous studies have correlated altered ER-mitochondrial connectivity to alterations in calcium transfer between both organelles, through overexpression or knockdown of proposed tethers, including MFN2 and VAPB-PTPIP51, or regulators thereof [28, 29, 34, 97] (Fig. 4). Several modulators of IP3R channel activity are present at or recruited to MAMs under various conditions. For example, various ER-resident chaperones and oxidoreductases can affect the exchange of calcium between the ER and mitochondria [96]. Calnexin (CNX) modulates local calcium levels by acting as a calcium buffer and through regulating the activity of IP3R and the sarco/endoplasmic reticulum calcium ATPase (Serca), the main ATPase pumping cytosolic calcium into the ER [52, 68, 90]. CNX targeting to the MAM is dependent on palmitoylation [68], and on the cytosolic sorting protein PACS2 [79] and Rab32 [16] two trafficking molecules that both regulate the composition of the MAMs (Fig. 4). The ER-resident Sigma1R is released from Bip upon ER calcium release, and binds to IP3Rs at the MAMs, in this way preventing their proteasomal degradation and sustaining prolonged Ca^2+^ uptake by mitochondria [46] (Fig. 4). Especially in neurons, both Sigma1R and PACS2 play a crucial role in mediating calcium homeostasis and loss of Sigma1R results in neuromuscular defects [47]. This is underscored by the fact that alterations in PACS2 and Sigma1R levels are associated with the pathogenesis of AD, ALS and FTD (see Table 1; Boxes 1, 2). In addition, presenilin mutations in AD are associated with an increased IP3R calcium release (see Table 1; Box 1). Finally, in Huntington’s disease ER-mitochondrial calcium transfer appears affected as well. Mutant huntingtin, but not the wild type protein, was found to interact with IP3R and facilitate calcium transfer between the ER and mitochondria [12, 83].

Calcium transfer to mitochondria occurs under both physiological and stress conditions. A moderate mitochondrial matrix calcium increase stimulates ATP synthesis through calcium dependency of metabolic enzymes in the Krebs cycle [87]. Functional coupling of the ER and mitochondria therefore stimulates aerobic metabolism, in response to a demand of ATP-requiring processes in the cytosol as well as in the ER [96, 111]. Hence, loss of contact has adverse effects on ER homeostasis, protein folding and energy metabolism [14], and will affect cell survival and proliferation, especially in cells with high-energy demands such as neurons. Mitochondrial calcium overload on the other hand is a potent inducer of the mitochondrial permeability transition pore and can thereby lead to OMM rupture and the escape of pro-apoptotic factors such as cytochrome c into the cytosol [87]. In this respect, regulation of ER-mitochondrial calcium transfer at the MAMs is essential in the switch between cell survival and death during various stress responses. In the context of Huntington’s disease for example, the binding of mutant huntingtin to IP3R may result in a heightened susceptibility of mitochondria to activate the permeability transition pore and the subsequent release of pro-apoptotic factors [23]. Intriguingly, proteins that modulate calcium transfer between mitochondria and the ER are affected in many neurodegenerative diseases and could be tightly linked to a loss in neuronal survival [94] (see Table 1).

### Regulation of mitochondrial dynamics and homeostasis

**Fig. 5:**
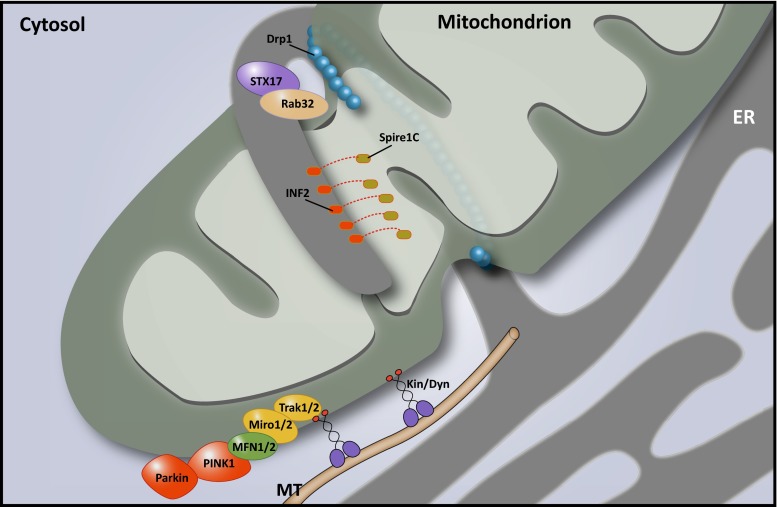
MAMs control mitochondrial dynamics. Endoplasmic reticulum (ER)-mitochondria contact sites are involved in mitochondrial fission and transport of mitochondria along cytoskeletal tracks. During fission, mitochondria-associated membranes (MAMs) determine the site of scission by contributing to the mitochondrial constriction required for dynamin-related protein 1 (Drp1) assembly. This constriction is accomplished through the actin-modulating activity of Spire1C and inverted forming 2 (INF2) at the membrane contact sites (MCSs). At least two other MAM proteins, syntaxin 17 (STX17) and Rab32, contribute to the regulation of mitochondrial fission by controlling Drp1 activity. Mitochondrial transport is primarily mediated by the mitochondrial Rho GTPases 1 and 2 (Miro1/2), which connect the mitochondria to motor proteins such as kinesins through the trafficking kinesin protein (TRAK) adaptor proteins. Miro depends on mitofusin1 and 2 (MFN1/2) to facilitate transport, and PTEN-induced putative kinase 1 (PINK1)-Parkin-dependent ubiquitination of Miro1/2 and MFN2 blocks mitochondrial movement. In zones of high calcium concentrations, such as those that occur at MAMs, calcium binding to the Miro1/2 EF-hand motifs releases the mitochondria from the cytoskeleton and halts their migration. The P56S mutation in VAMP-associated protein B and C (VAPB), which results in a higher degree of ER-mitochondria contact and calcium crosstalk, consequently results in axonal transport defects of mitochondria

The function of both the ER and mitochondria strongly depends on a highly regulated balance in membrane dynamics. Mitochondrial dynamics include their transport along cytoskeletal tracks throughout the cell, in addition to regulated fission and fusion. Combined, these dynamics ensure optimal mitochondrial metabolism and, in neurons, the correct distribution of mitochondria to dendrites and synapses. Especially in neurons with long axons this poses a challenge, which is highlighted by the many diseases caused by defects in the proteins involved in mitochondrial dynamics [19] (see Boxes 2–4; Table 1). Mitochondrial fission is known to require the assembly of dynamin-related protein 1 (Drp1) in a helix around the OMM, followed by constriction of this ring (Fig. 5). However, it has long been unclear what signaling events precede this process and mark the site of Drp1 assembly. In 2011, Friedman et al. showed in Cos7 cells that focal accumulation of Drp1 and subsequent fission occurs at or near sites of contact with the ER, and the same holds true for the yeast Drp1 ortholog Dnm1 [37]. Mitochondrial constriction occurs in the vicinity of the ER even in the absence of Drp1 and the mitochondrial fission factor (Mff). These observations suggested that ER tubules mark the sites of mitochondrial fission and induce initial constriction, followed by the recruitment of Mff and finally Drp1 helix assembly that mediates the division [37]. Indeed, recent studies have described that actin polymerization, induced at the MCSs by an ER-bound isoform of Inverted Formin 2 (INF2), is capable and required to induce the initial constriction required for mitochondrial fission (Fig. 5) [58]. Spire1C, a newly described mitochondrial isoform of the actin-nucleating Spire proteins, interacts with INF2 to specifically nucleate actin filaments only at sites where the ER and mitochondrial membranes are closely apposed. Loss of this interaction results in long tubular mitochondria reminiscent of defective fission [70]. Mitochondrial constriction prior to Drp1 assembly depends on this actin polymerization step, however, additional force generated by Myosin II may also contribute to this process [30, 57]. How mutations in INF2, causing Charcot–Marie–Tooth disease type 2D and glomerulopathy [15] affect this process remains to be determined. Finally, it was shown in a very elegant way that Drp1 activity at the MAMs is strictly regulated by phosphorylation in a process that involves Rab32 and syntaxin 17 (STX17) [4] (Fig. 5). This process seems tightly linked to the regulation of calcium homeostasis and the initiation of autophagy (see further). In addition, SUMOylation of Drp1 was reported to affect its function at the MAM, stabilizing the ER-mitochondria contact site and thus promoting calcium crosstalk and cytochrome c release [86].

In contrast to the role of Gem1p in ER-associated mitochondrial division in yeast, no such role has been described for the mammalian Gem1 orthologs Miro1 and Miro2. In mammalian cells, both Miro proteins 1 and 2 are known as essential regulators of mitochondrial motility [36, 69, 92]. These atypical Rho GTPases are anchored in the OMM and mediate anterograde and retrograde transport by connecting the mitochondria to microtubule (MT)-bound kinesin and dynein through the trafficking kinesin (TRAK1 and TRAK2) adaptor proteins (Fig. 5) [36, 69]. Neuron-specific loss of Miro1 in mice results in a neuronal disease phenotype that most closely resembles that of spastic paraplegia patients [80] (see Box 4; Table 1).

Interestingly, several observations point to a role of MAMs in Miro1/2-mediated cellular transport of mitochondria. First, as mentioned, the yeast ortholog of Miro1/2 is a regulatory subunit of the ERMES complex and Miro1 localizes at ER-mitochondria contact sites in mammalian cells [56]. Second, at sites of close contact, the ER and mitochondria move along acetylated MTs while staying attached, suggesting coordinated transport [38]. Third, in addition to coordinating fusion and tethering to the ER of the OMM, MFN2 is required for axonal transport and both MFN1 and MFN2 interact with Miro1/2 [75]. Fourth, mitochondrial transport is regulated by calcium through the EF-hand motifs in Miro1/2, which function as a calcium sensor. Calcium binding of Miro1/2 induces a conformational change that disconnects the mitochondria from the MT track it moves along, thus halting its transport [20, 69, 92]. Due to the relatively low affinity of Miro1/2 to calcium, calcium-dependent halting of mitochondrial transport is most likely to occur at calcium hotspots, such as the ER-mitochondria MCSs [20, 92]. The dependence on high calcium regions indicates a mechanism whereby the proximity with the ER can determine the redistribution of mitochondria. This provides the cell with a powerful system of targeting mitochondria based on local energy requirements. Particularly in neurons, this might be a relevant factor in determining the distribution of mitochondria to the dendrites, synapses and the nodes of Ranvier that is currently insufficiently understood. That such a system is essential for neuronal survival is underscored by the many reports of mitochondrial transport defects in neurodegenerative disorders [19]. Much research has focused on the connection between mitochondria and MTs in this context. It is becoming increasingly clear, however, that the connection with the ER is also a major determinant for axonal transport of mitochondria (see Boxes 2–4).

### MAMs as a potential regulator of autophagy and mitophagy

**Fig. 6:**
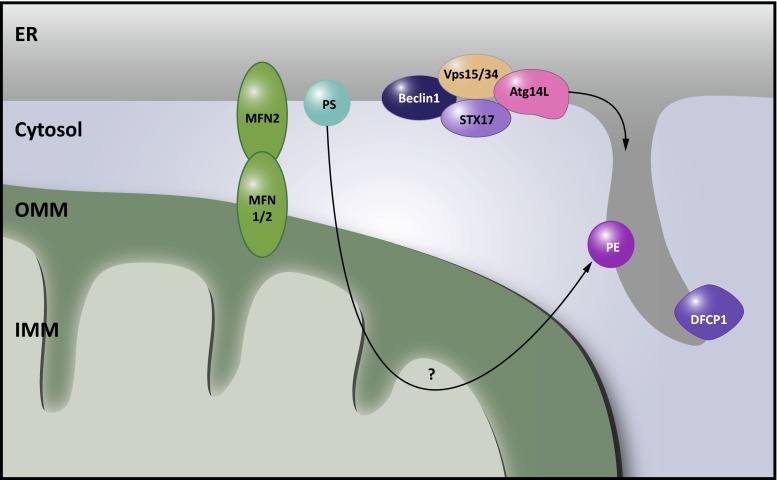
MAMs as sites for autophagosome biogenesis. Mitochondria-associated membranes (MAMs) were identified as a membrane source for autophagosome biogenesis. In a first instance, phosphatidylethanolamine (PE) synthesis at MAMs was believed to be a crucial lipid source for these emerging organelles. However, ER-mitochondria contact sites appear to play a more direct role. Syntaxin 17 (STX17) at MAMs recruits early autophagosome markers including Atg14L, Vps15, Vps34 and Beclin1. In addition, the omegasome marker double FYVE-containing protein 1 (DFCP1) translocates to these sites upon autophagy induction. Underscoring the relevance of ER-mitochondria membrane contact sites (MCSs), loss of phosphofurin acidic cluster sorting protein 2 (PACS2) or mitofusin 2 (MFN2) abrogates autophagosome biogenesis

Macroautophagy is the process whereby damaged proteins and organelles are cleared from the cell by sequestering them in a double membrane-bound vesicle termed the autophagosome. Subsequent delivery to the lysosome allows proteasomal breakdown and recycling of the substrates. Debate is still ongoing with respect to the origin of the phagophore and the identity of membrane donor sources needed for its expansion to form the mature autophagosome [62]. Axe et al. identified the omegasome, a phosphatidylinositol-3-phosphate-enriched membrane structure attached to the ER, as a membrane source for phagophore development [8]. In addition, the mitochondria, harboring many autophagy-related (ATG) proteins and regulators thereof, have been proposed as an origin for phagophore formation, based on the transfer of a fluorescently labeled mitochondrial marker to the phagosome [43]. Intriguingly, several ATG proteins have been found to accumulate specifically at ER-mitochondria MCSs in conditions of starvation, and more and more evidence is accumulating that MAMs might be the actual site of autophagosome formation, unifying both earlier models [43]. Hailey et al. first speculated on the requirement of lipid transfer from the ER to mitochondria during autophagosome biogenesis. In mouse cells lacking MFN2, autophagy induction was disturbed. The authors contributed this to a decreased lipid transfer towards mitochondria as a consequence of ER-mitochondrial uncoupling and concluded that lipids transferred from the ER accumulate in the OMM, from where they are trafficked to the expanding phagophore [43] (Fig. 6). A more direct involvement of MAMs in autophagosome biogenesis was proposed by Hamasaki and coworkers [44]. Upon starvation of mammalian cells, Vps15, Vps34, Atg14L, Beclin1—early markers of phagophore formation—and the omegasome marker double FYVE-containing protein 1 (DFCP1) accumulate in the MAM fraction (Fig. 6) [44]. This might be initiated by early translocation of STX17 [4]. In starvation conditions, STX17 translocates in an mTOR/ULK dependent manner to a domain of ER-mitochondrial contact resistant to digitonin, where it recruits ATG14L [4]. ATG14L then interacts with the phosphoinositide 3-kinase complex consisting of Beclin1, p150 and Vps34, resulting in local phosphatidylinositol-triphosphate production. This alters the local lipid composition of the MAM membrane, which leads to the recruitment of WD-repeat domain phosphoinositide interacting (WIPI) proteins, the effector proteins and the mediators of autophagosome biogenesis. It has been proposed that this could be sufficient to induce a deformation of the membrane [62]. Consistent with a role for the MAMs in autophagosome formation, knockdown of MFN2 or PACS2 abolished ATG14L puncta formation and downstream LC3 lipidation in starved cells, and caused defective STX17 localization at the MAM [44].

In addition, during the selective autophagy of mitochondria, known as mitophagy, ER-mitochondria contact sites appear to constitute a platform promoting mitophagosome formation. In yeast, efficient mitophagy depends on coupling of mitochondria and the ER through ERMES, while bulk autophagy can apparently operate in the absence of this tether [13]. ERMES colocalizes with sites of mitophagosome biogenesis and affects the formation of the phagophore that engulfs the mitochondria destined for degradation [13, 71].

In mammalian cells, mitochondria destined for degradation recruit the E3 ubiquitin ligase Parkin to the OMM through PTEN-induced putative kinase 1 (PINK1) kinase activity. Ubiquitination of Parkin substrates subsequently recruits the autophagy machinery [31]. ER-mitochondria MCSs are implied in this process in several ways. First, focal accumulation of DFCP1 along mitochondria labeled for degradation with exogenously expressed Parkin suggests a role for the ER/MAM as a membrane source for the mitophagosome [115]. Second, Drp1-mediated fission, at least in part mediated by contacts with the ER [37], is suggested to promote mitophagy by breaking off pieces of mitochondria promoting their engulfment by the mitophagosome [105]. Lastly, depolarization of the IMM through calcium (over)loading could be involved in PINK1 and Parkin translocation to the OMM, activating mitophagy [31, 53].

While the exact mechanisms governing autophagy induction and progression are not entirely clear, several lines of evidence point to an essential role of the ER-mitochondria interface during this process. The presence at the MAMs of several important players in metabolism, such as mTORC2 [11], allows to speculate that ER-mitochondria MCSs could represent a site of crosstalk between sensors of the cells’ energetic needs and the early players in autophagy. Additionally, through interorganelle communication, dysfunctional mitochondria may be sensed at the MAMs, followed by mitophagy induction and targeted removal of these unhealthy mitochondria. Future research is needed to study these mechanisms and how they are disrupted in disease. Dysregulation of neuronal energy metabolism and mitochondrial homeostasis are important factors contributing to neurodegeneration [94]. This appears relevant in particular for PD (see Box 3), however, autophagy is a process whose dysregulation is widely implied in neurodegenerative disorders [73].

## Conclusion

MCSs provide pathways of intracellular signaling that are only beginning to be unraveled. Although many questions remain as to how ER-mitochondria MCSs are maintained and regulated, it is clear that many different pathways intertwine at these signaling hubs. Whether all the different functions described here occur simultaneously at the same MCS, or whether specialized ER-mitochondria MCSs exist that mediate a subset of these processes is currently unclear. Gaining further insight into the protein tethers that connect both organelles will be crucial to shed more light on the regulation of MAM functions.

As these functions become uncovered, also the relevance of ER-mitochondria contact sites in disease becomes apparent. Neuronal cells in particular appear to be very vulnerable to insults affecting the balance in ER-mitochondrial communication. Indeed, many of the processes that require these contact sites are implied in neurodegeneration. As such, resolving the role of MAMs in the pathomechanisms leading to these disorders may unify several of the previously identified defects that occur during neurodegeneration, including imbalances in calcium or lipid signaling, mitochondrial dynamics and autophagy pathways. Since tipping the balance in either direction appears detrimental for neuronal survival, uncovering the different players that maintain this delicate equilibrium will be crucial to understand the role that MCSs play in these disorders and possibly lead to novel strategies to reverse ER-mitochondrial homeostasis defects in patients.

## Box 1: Alzheimers’s dementia

Aberrant cleavage of the amyloid precursor protein (APP) is the main hallmark of AD pathogenesis. Mutations in PS1 and PS2, components of the ɣ-secretase complex involved in APP processing, are major causes of familial AD (FAD) (Table 1). Consistent with their known association with lipid rafts, PS1, PS2, APP and ɣ-secretase activity are enriched at MAMs [6]. Moreover, in cells deficient of MFN2, which showed diminished MAM function in terms of lipid metabolism, the enzymatic activity of the ɣ-secretase was reduced by approximately 50 %, showing that APP processing relies on intact ER-mitochondria crosstalk [5]. Vice versa, MAM function also depends on PS1 and PS2. ER-mitochondria MCSs and MAM-dependent lipid metabolism were shown to be upregulated in cells deficient of PS1 and PS2 as well as in cell models of both familial and sporadic AD (SAD) [5]. The complicated interplay between the ɣ-secretase constituents and activity and ER-mitochondrial crosstalk also extends to calcium signaling. The PS1 and PS2 proteins were shown to interact with IP3Rs and FAD-associated mutations in either gene result in a higher IP3R gating activity and exaggerated calcium release from the ER, followed by increased APP cleavage [21, 22]. In turn, exposing hippocampal neurons or neuroblastoma cells to amyloid β enhances ER-mitochondria contact and calcium transfer [33, 47]. Post-mortem analysis of human AD brain and those of AD mouse models showed altered expression levels of PACS2 and Sigma1R, MAM proteins involved in calcium handling, thus further establishing dysregulated MAM signaling as a hallmark of AD [47]. How exactly MAM function influences APP processing and how a defect in the ɣ-secretase in turn affects ER-mitochondria MCSs remains to be further clarified. The finding that MAMs are involved early in the pathogenesis of both FAD and SAD is a major breakthrough nonetheless, as it can explain many of the different neuronal defects associated with AD, including calcium homeostasis, mitochondrial dysfunction, oxidative stress and lipid metabolism [93]. Most recently, it was also reported that the E4 allele of the apolipoprotein E (ApoE4), a major risk factor for developing SAD, contributes to increased crosstalk at the ER-mitochondria MCS [101]. MAMs are thus placed center-stage in the AD pathogenesis and therefore make for a promising target for therapeutics. One such target could be GSK3β, a central regulator of homeostasis in the brain that is implied in a large number of disorders including AD [66]. As further elaborated upon in BOX 2, GSK3β was recently identified as a negative regulator of ER-mitochondria MCSs formation [97].

## Box 2: Amyotrophic lateral sclerosis

MAM integrity and signaling have been found affected in several ALS subtypes. VAPB is an ER protein involved in tethering the MAM to mitochondria [29]. A point mutation in the gene encoding VAPB was identified in patients suffering from the severe familial ALS type 8 [81] (Table 1). Mutant VAPB is enriched at the MAM, where increased binding with PTPIP51 tightens the connection at the ER-mitochondria interface and results in higher mitochondrial calcium peaks (Fig. 4) [29]. More recently, two other proteins involved in ALS have been identified as modulators of this interaction: glycogen synthase kinase-3β (GSK3β), established as a negative regulator of VAPB/PTPIP51 interactions, and TAR DNA-binding protein 43 (TDP43), which activates GSK3β [97] (Figs. 2, 4). GSK3β hyperactivity is not only connected to ALS, but it also takes a central role in the disease mechanisms leading up to AD and PD, both in familial and sporadic cases [41, 66] (Table 1). TDP43 accumulation on the other hand is a hallmark of the FTD/ALS pathology and mutations in the TDP43 gene occur in approximately 3 % of patients with familial ALS as well as in 1.5 % of sporadic cases [63] (Table 1). Overexpression of TDP43 leads to an activation of GSK-3β and consequently weakens ER-mitochondria coupling [97]. In a transgenic TDP43 mouse model for ALS, motor neurons show reduced contact between the ER and mitochondria. Similarly, overexpression of wild type or mutant TDP43 in NSC34 cells disrupts both the structural and the functional connection between both organelles, ensued by a diminished exchange of calcium [97]. The fact that in one ALS subtype calcium signaling at MAMs is found to be upregulated, whereas it appears downregulated in another, underscores the importance of balanced communication between both organelles for neuronal survival.

In addition to tightened contact between the ER and mitochondria and elevated calcium transfer, expression of VAPB P56S disrupts the connection of Miro1 to tubulin, obstructing anterograde, but not retrograde mitochondrial transport in rat cortical neurons [77]. This phenotype was rescued by the co-expression of a calcium-insensitive Miro1 mutant, emphasizing the importance of controlled calcium signaling at MAMs for mitochondrial transport [77]. Of note, an earlier report showed that fibroblasts derived from VAPB P56S patients as well as neuronal cultures displayed intracellular aggregates containing VAPB and Sigma1R, a major regulator of calcium trafficking at MAMs [46]. Moreover, pharmacological Sigma1R activation was neuroprotective in this context and led to decreased VAPB aggregation [85]. Interestingly, mutations in the gene coding for Sigma1R are also associated with ALS/FTD [2, 67]. A physiological connection between VAPB and Sigma1R at the MAMs remains to be determined, however, the defect in mitochondrial dynamics upon loss of Sigma1R points to a shared function of both proteins in ER-regulated axonal transport of mitochondria [9]. As the large majority of ALS cases are sporadic rather than familial, it will be crucial to study whether MAMs also play a role in these sporadic cases.

## Box 3: Parkinson’s disease

Parkinson’s disease is caused by loss of dopaminergic neurons in the substantia nigra. A minority of PD cases are monogenic, caused by mutations in the genes encoding Parkin, PINK1, alpha-Synuclein (α-Syn) or the protein deglycase DJ-1 amongst others [94]. Parkin and PINK1, as mentioned, play important roles in mitophagy and mutations in the genes encoding these proteins are believed to disrupt the proper degradation of defective mitochondria [31]. The function of Parkin, however, appears broader than merely marking damaged mitochondria. In cultured rat neurons, glutamate excitotoxicity elicits the accumulation of Parkin at mitochondria, the ER and the ER-mitochondrial interface, without inducing mitophagy [107]. Overexpression of Parkin was previously shown to increase physical and functional coupling of the ER and mitochondria, stimulating ATP production and calcium exchange, while knockdown has the opposite effect and disrupts mitochondrial morphology [17]. The exact function of Parkin at the ER or its junctions with mitochondria and whether and how this is connected to its function in mitophagy is currently unclear, however, these observations point to a protective role for Parkin during excitotoxic stress, by maintaining ER-mitochondrial crosstalk.

Like Parkin, also α-Syn and DJ-1 overexpression promotes MAM function and interaction with mitochondria (Fig. 2) [18, 42, 82]. Intriguingly, all these proteins were reported to interact with Grp75, providing a plausible mechanism whereby they can affect ER-mitochondria calcium signaling, although alternative pathways may be involved as well [51]. Wild type α-Syn, known to have a high affinity for lipid rafts, localizes to MAMs where it promotes physical contact with mitochondria [18]. Consistent with a role for α-Syn in contact formation, loss of α-Syn at the MAMs results in diminished ER-mitochondria signaling [18, 42]. In contrast to wild type α-Syn, PD-causing mutant forms of α-Syn downregulate ER-mitochondrial apposition, presumably through disrupted interaction with lipid rafts in the case of A30P mutant α-Syn, or through decreased levels of total α-Syn in the case of the A53T mutant [5, 35]. Accumulation of α-Syn in protein aggregates termed Lewy bodies, a major hallmark of both familial and sporadic PD, could therefore represent of loss of function of this protein at the MAMs, which may underlie the disorder.

No direct involvement for PINK1 in ER-mitochondrial crosstalk has been reported. It does, however, play a role in mitochondrial motility, regulation of which also takes place at this interface. In addition to local calcium levels, mitochondrial motility is regulated through proteasomal breakdown of the transport complex, as PINK1 promotes the proteasomal degradation of Miro1/2 and MFN1/2 in a Parkin-dependent manner [65, 102, 113]. Intriguingly, loss of Miro was shown to rescue the phenotype caused by expression of PD-causing PINK1 mutations in Drosophila and facilitates clearance of damaged mitochondria in HeLa cells, suggesting that this process plays a significant role in the disease [65]. Further research is needed to investigate how contacts of mitochondria with the ER are involved in this pathway and whether ubiquitination of MFN2 by Parkin can modulate these contacts, as reported for MITOL [98]. Combined, these findings accentuate the complex interdependence of mitochondrial quality control and mitochondrial dynamics, pathways that clearly intersect at the ER-mitochondrial interface.

## Box 4: Axonopathies

CMT and HSP are both caused by degeneration of long axons, in the case of CMT those of the peripheral motor and/or sensory nerves, while the upper motor neurons are predominantly affected in the case of HSP. Unsurprisingly, these disorders share many commonalities with regards to the pathomechanisms involved and a disproportionate number of affected genes localizes to mitochondria, the ER, or the MCSs shared by both [103]. In HSP, membrane shaping of the ER is a major theme, with involved genes including Receptor Expression-Enhancing Protein 1 and 2 (REEP1/2), Spastin, Reticulon 2 (RTN2) and Atlastin 1 (ATL1). ATL1 and ATL3 are also found mutated in hereditary sensory and autonomous neuropathy (HSAN) [49, 54]. Work in yeast suggests that defects in ER-shaping proteins may affect ER-mitochondrial MCSs and lipid transfer. Interestingly, the RTN2 homolog RTN4b was identified as a negative regulator of crosstalk between both organelles [99]. More recently, HSP-causing mutations in or loss of REEP1 were also reported to disrupt ER-mitochondria MCSs [64]. Though no direct effect on MCSs was reported, neurons differentiated from patient-derived induced pluripotent stem cells carrying the HSP-causing P342S mutation in ATL1 display not only defects in ER morphology, but also in the axonal transport of mitochondria [116]. Another example of an ER-bound protein that modulates mitochondrial dynamics is INF2, mutations in which cause CMT associated with glomerulopathy [15]. INF2 modulates actin dynamics required for MAM-dependent mitochondrial fission (see main text and Fig. 5) [58, 70]. How CMT-causing mutations in this gene affect mitochondrial dynamics remains to be elucidated, however, it is clear that ER-resident proteins can play significant roles in mitochondrial dynamics through the interface between both organelles. Similarly, proteins known for their role in mitochondrial dynamics can affect the ER network. CMT-causing mutations in MFN2 disrupt ER morphology and contact with mitochondria [28], in addition to interfering with mitochondrial dynamics and distribution within axons [75, 76]. Since these mutations do not affect the interaction of MFN2 with Miro1/2, aberrant connectivity with the ER might be the underlying factor causing mislocalization of mitochondria within axons [76]. It is clear that the interplay between ER and mitochondrial network dynamics and interorganelle communication plays a pivotal role in axonal survival and degeneration. Though the exact mechanisms controlling mitochondrial motility at the MCSs with the ER are the subject of ongoing research, several observations suggest that a multitude of pathways can impinge on this process and it is likely that the signaling hub where these pathways are integrated, are the ER-mitochondria contact sites.

## Electronic supplementary material


**Supplementary movie 1: 3D morphology of ER-mitochondria contact sites.** The same dataset and reconstruction as described in Fig. 1 are shown. A part of the original electron micrographs obtained using the Zeiss Auriga Crossbeam microscope is shown by moving along the Z-axis. Next, the volume is shown by the reverse movement along the Z-axis and the simultaneous rotation around the X-axis while the rendered reconstruction of mitochondria (green) and their associated endoplasmic reticulum (ER) (magenta) appear. Next, an XZ-projected image appears while zooming in on one ER-mitochondria contact site. This contact site is shown in 3D as the image stack is digitally resliced along its Y-axis. IMOD was used for image registration, manual segmentation, 3D rendering and animation. (AVI 102184 kb)

## Abbreviations

AD: Alzheimer’s dementia
ALS: Amyotrophic lateral sclerosis
APP: Amyloid precursor protein
ATG: Autophagy-related
ATL1/3: Atlastin 1/3
CMT: Charcot–Marie–Tooth disease
CNX: Calnexin
DFCP1: Double FYVE-containing protein 1
DJ-1: Protein deglycase DJ-1
Drp1: Dynamin-related protein 1
EMC: Endoplasmic reticulum membrane complex
ER: Endoplasmic reticulum
ERMES: Endoplasmic reticulum-mitochondria encounter structure
FAD: Familial Alzheimer’s dementia
FIB-SEM: Focused ion beam scanning electron microscopy
Fis1: Fission 1 homolog
FTD: Frontotemporal dementia
GSK3β: Glycogen synthase kinase-3β
HSAN: Hereditary sensory and autonomous neuropathy
HSP: Hereditary spastic paraplegia
IMM: Inner mitochondrial membrane
INF2: Inverted formin 2
IP3R: Inositol 1,4,5-triphosphate receptor
MAM: Mitochondria-associated membrane
MCS: Membrane contact site
MCU: Mitochondrial calcium uniporter
Mff: Mitochondrial fission factor
MFN1/2: Mitofusin 1/2
miro1/2: Mitochondrial Rho GTPases 1/2
MT: Microtubule
OMM: Outer mitochondrial membrane
PA: Phosphatidic acid
PACS2: Phosphofurin acidic cluster sorting protein 2
PC: Phosphatidylcholine
PD: Parkinson’s disease
PE: Phosphatidylethanolamine
PEMT: Phosphatidylcholine-*N*-methyltransferase
PINK1: PTEN-induced putative kinase 1
PS1/2: Presenilin 1/2
PS: Phosphatidylserine
Pss1/2: Phosphatidylserine synthase 1/2
PTPIP51: Protein tyrosine phosphatase-interacting protein 51
REEP1/2: Receptor expression-enhancing protein 1/2
RTN2/4b: Reticulon 2/4b
RyR: Ryanodine receptor
SAD: Sporadic Alzheimer’s dementia
Sigma1R: Sigma non-opioid intracellular receptor 1
STX17: Syntaxin 17
TDP43: TAR DNA-binding protein 43
TEM: Transmission electron microscopy
Tom5: Outer mitochondrial membrane translocase complex 5
TRAK1/2: Trafficking kinesin protein 1/2
VAPB: Vesicle-associated membrane protein-associated protein B
VDAC1: Voltage-dependent anion channel 1
α-Syn: Alpha-synuclein
